# Road Accident due to a Pancreatic Insulinoma: A Case Report

**DOI:** 10.1097/MD.0000000000000537

**Published:** 2015-03-27

**Authors:** Amilcare Parisi, Jacopo Desiderio, Roberto Cirocchi, Veronica Grassi, Stefano Trastulli, Francesco Barberini, Alessia Corsi, Alban Cacurri, Claudio Renzi, Fabio Anastasio, Francesca Battista, Giacomo Pucci, Giuseppe Noya, Giuseppe Schillaci

**Affiliations:** From the Unit of Digestive and Liver Surgery (AP, JD, VG, ST, AC), Santa Maria Hospital, Terni; Department of General and Oncologic Surgery (RC, FB, AC, CR, GN), University of Perugia, Perugia; Unit of Internal Medicine (FA, FB, GP, GS), Santa Maria Hospital, Terni; and Department of Medicine (FA, FB, GP, GS), University of Perugia, Perugia, Italy.

## Abstract

Insulinoma is a rare pancreatic endocrine tumor, typically sporadic and solitary. Although the Whipple triad, consisting of hypoglycemia, neuroglycopenic symptoms, and symptoms relief with glucose administration, is often present, the diagnosis may be challenging when symptoms are less typical.

We report a case of road accident due to an episode of loss of consciousness in a patient with pancreatic insulinoma. In the previous months, the patient had occasionally reported nonspecific symptoms. During hospitalization, endocrine examinations were compatible with an insulin-producing tumor. Abdominal computerized tomography and magnetic resonance imaging allowed us to identify and localize the tumor. The patient underwent a robotic distal pancreatectomy with partial omentectomy and splenectomy.

Insulin-producing tumors may go undetected for a long period due to nonspecific clinical symptoms, and may cause episodes of loss of consciousness with potentially lethal consequences. Robot-assisted procedures can be performed with the same techniques of the traditional surgery, reducing surgical trauma, intraoperative blood loss, and hospital stays.

## INTRODUCTION

Insulinomas are the most common functional variety of neuroendocrine pancreatic tumors.^1^ Whipple triad of hypoglycemia, neuroglycopenic symptoms, and symptoms relief with administration of glucose is often present. Neuroglycopenic symptoms include dizziness, confusion, apathy, amnesia, personality and behavioral changes, visual disturbances, seizure, and coma.^2^ The diagnosis of insulinoma is often delayed due to its relative rarity, and variable clinical presentation, which often includes nonspecific symptoms. The suspicion of insulinoma and the beginning of biochemical investigation is often delayed by a mean of 3.8 years.^3^ It is diagnosed by clinical, biochemical, and imaging investigations. Surgical resection is the curative treatment.^4^ We report a case of a car accident due to loss of consciousness caused by a pancreatic insulinoma.

## CASE REPORT

A 42-year-old man (the patient had given consent) was admitted to the hospital for a motor vehicle accident due to loss of consciousness. The patient reported sudden loss of consciousness while driving his car. He was completely amnesic for the episode, and had not reported similar episodes before. Medical history included a β-thalassemic trait. No diabetes, ischemic heart disease, or cerebrovascular disease was reported. The patient reported occasional episodes of weakness, sweating, pallor, tremor, confusion, paresthesias, and visual blurring in the previous year. He denied taking any drugs. Cardiovascular, respiratory, and neurological examinations were normal. On presentation, the Glasgow Coma Scale was ≥12 with normal vital signs. Psychomotor activity was normal; perception and cognitive functions were normal. A brain computerized tomography (CT) scan was normal. Laboratory tests were normal except a low fasting blood glucose level. For this reason, the patient underwent a 72-hour fasting test, which was stopped after 6 hours because of a blood glucose level of 29  mg/dL; during the test blood glucose level remained low and serum insulin level was elevated (33.6 μU/mL).

**FIGURE 1:**
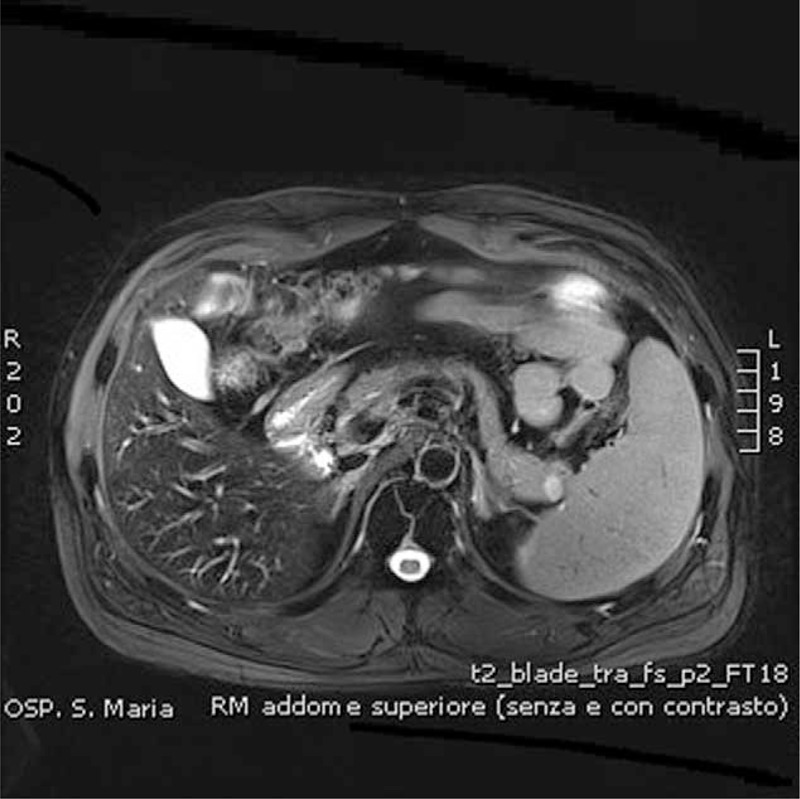
Abdominal magnetic resonance imaging.

**FIGURE 2:**
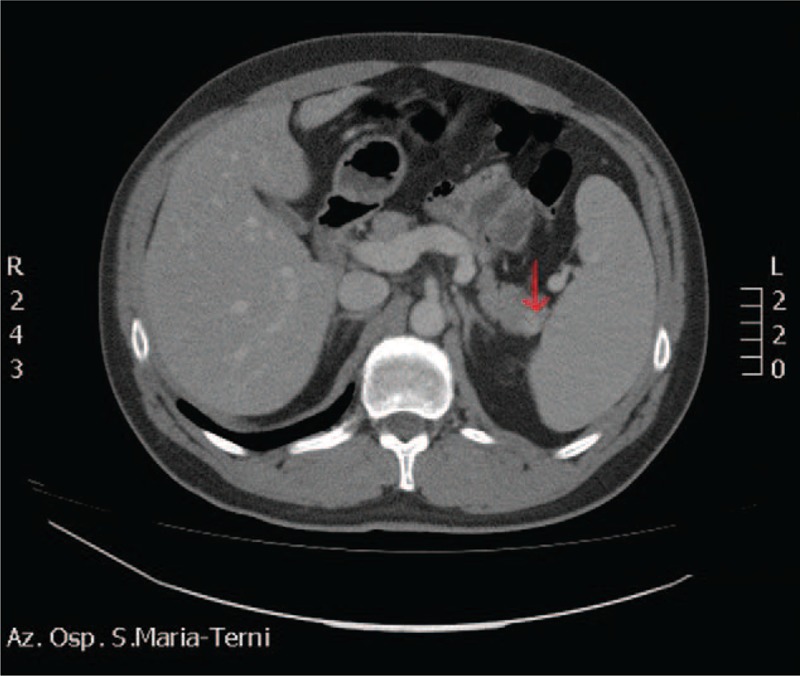
Abdominal computerized tomography scan: solid 11-mm nodule in the tail of the pancreas (arrow).

Endocrine tests excluded other causes of hypoglycemia, such as hypopituitarism and adrenal insufficiency. An abdominal magnetic resonance scan showed an 11-mm nodule of the tail of the pancreas, a normal pancreatic duct, and no evidence of retroperitoneal lymphadenopathy (Figure 1). An abdominal contrast-enhanced CT confirmed the presence of a slightly hypervascular 11-mm solid nodule of the tail of the pancreas, with no other relevant findings (Figure 2). A diagnosis of pancreatic insulinoma was made, and the patient was transferred to the surgery unit for surgical treatment. Informed consent was obtained from the patient, then a robotic distal pancreatectomy with partial omentectomy and splenectomy was performed (Ethics Committee approval was not required in this case, since robotic surgery falls within common clinical practice in Italy for this indication).

### Surgical Procedure

**FIGURE 3:**
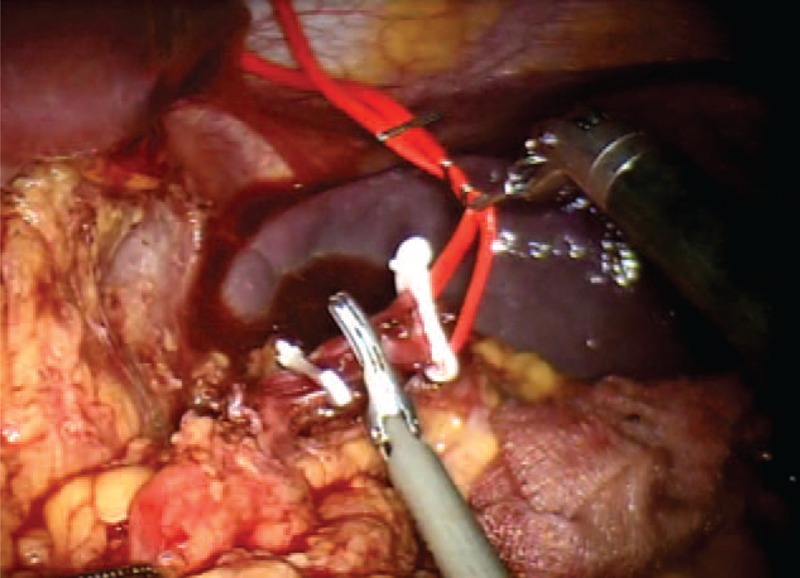
Section of the splenic artery after placement of hem-o-lok.

**FIGURE 4:**
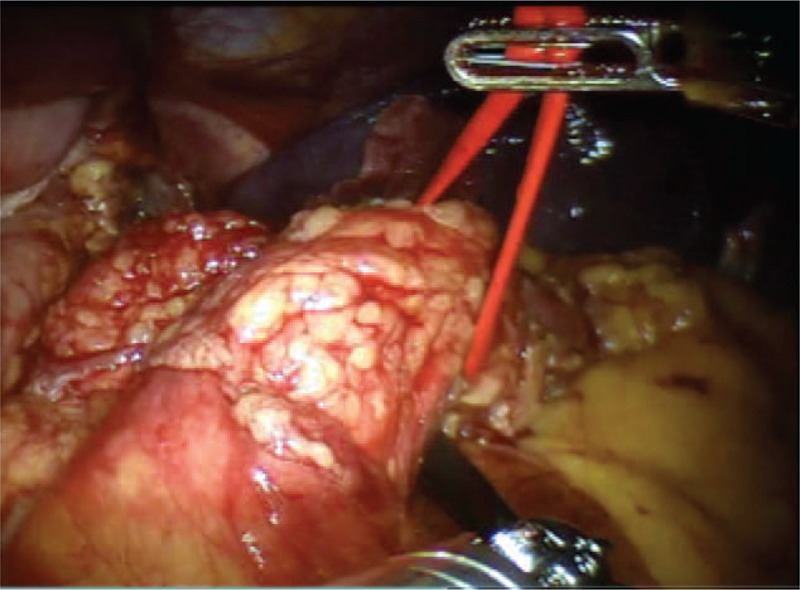
Isolation of the pancreas at the isthmus.

**FIGURE 5:**
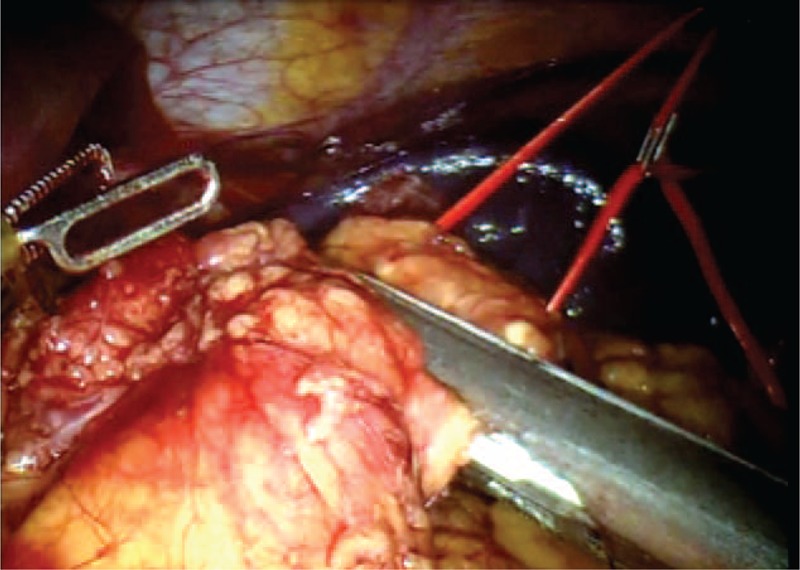
Section of the pancreas using mechanical stapler.

**FIGURE 6:**
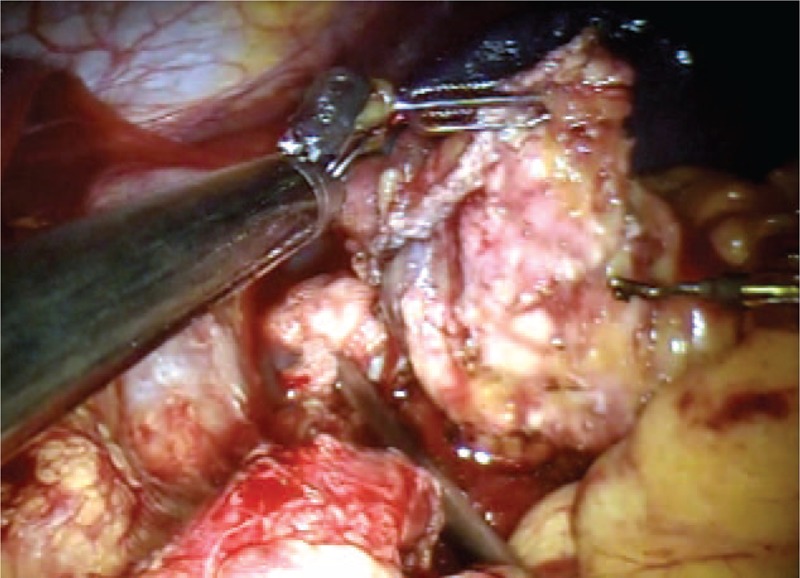
The dissection of the pancreas.

**FIGURE 7:**
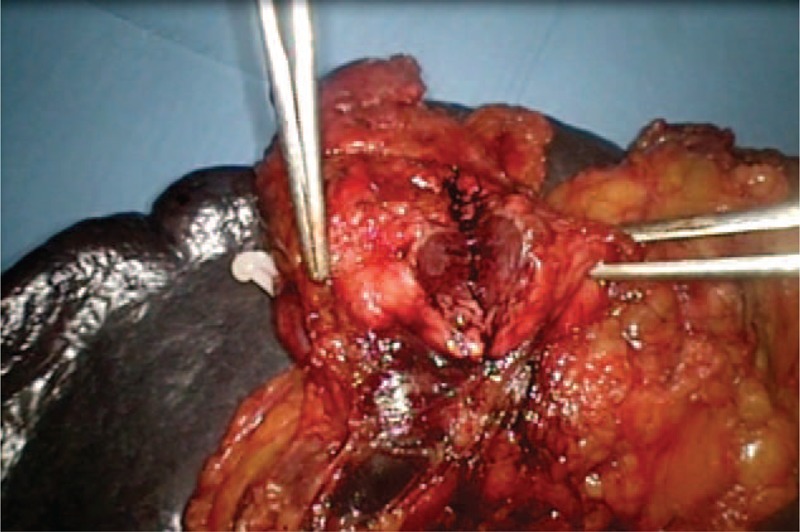
The tumor.

The patient is in dorsal position with the legs spread. One optical and 3 working robotic trocars are used. A 12 mm extra-port is also placed. It is used by the assistant surgeon in the various surgical phases to introduce the aspirator, mechanical stapler and stitches. Using atraumatic forceps, the stomach is grasped and lifted. A wide opening in the gastrocolic ligament is performed using the Ligasure system. The pancreas is exposed by elevating the stomach and pulling the colon downward with the atraumatic forceps. Once the access in the omental pouch is achieved, the dissection of the splenic artery is performed. The splenic artery is isolated and shown on a loop. Section is performed after placing a hem-o-lok ligation system (Figure 3). The pancreatic isthmus is identified, removed from the portal trunk at the level of the posterior wall, and surrounded by a loop (Figure 4). Section of the pancreas at the level of the isthmus is performed by robotics stapler (Figure 5), and after careful hemostasis the body and tail are dissected using the hook (Figure 6). After dissecting the splenic lower-pole vessels, the spleen is separated from its adhesions with the diaphragm and the stomach. The specimen is positioned in the endo-bag, and a peritoneal lavage is done. Hemostasis is achieved with cautery and compression. A drainage tube is placed in a retrogastric position. The specimen is removed from the abdomen through a 12-mm port (Figure 7). Surgical time was 210 minutes, and the blood loss was 90 mL; no transfusion was performed. Nasogastric tube, urinary catheter, and drainage tube were removed after 24 hours. On the first day, the pain on the Visual Analog Scale (VAS) score was equal to 3, and bowels were open to gas. On the second day, bowel sounds were audible and a liquid diet was started; on the third day, the bowels were open to the feces and a solid diet was started. On the fourth postoperative day, the patient was discharged in good health with proper glucose levels. Histopathological examination of tissues revealed a normal but enlarged spleen of 160 × 135 × 50 mm. In the resected caudal portion of the pancreas, a 14 × 20 mm brownish nodule, apparently capsulated, was present. The omentum included 2 capsulated, brownish red–colored nodules, respectively, 10 and 9 mm in diameter, compatible with accessory spleens. The histological diagnosis was well-differentiated neuroendocrine tumor (NET-G1), limited to the pancreas. No evidence of perineural and angiolymphatic neoplastic infiltration was present. Mitotic index was <1 mitotic figures per 10 high power fields. There was no infiltration of the surgical margins. The immunohistochemical analysis showed chromogranin +, synaptophysin +, NSE +, CD56 +/−, ki-67/MIB1 1%. After 90 days, all symptoms attributable to hypoglycemia disappeared, and plasma glucose and insulin levels were in the normal range.

## DISCUSSION

Insulinoma is a rare pancreatic endocrine tumor, typically sporadic and solitary. Over 90% of all insulinomas are benign. The Whipple triad of low blood glucose levels (below 50 mg/dL), hypoglycemia symptoms especially after fasting or heavy exercise, and dramatic reversal of central nervous system abnormalities by glucose administration is often present.^5^

Although patients with insulinoma have symptoms of hypoglycemia resulting from neuroglycopenia and increased catecholamine release, the diagnosis can be difficult in cases with incomplete functional activity. Blunted symptoms of hypoglycemia and negative laboratory investigations can retard the diagnosis,^6^ and symptoms may be misattributed to psychiatric, cardiac, or neurological disorders.^7^ In our case, the diagnosis was preceded in the previous year by several episodes of weakness, sweating, and pallor, which abated spontaneously or after having a snack. The above symptoms had been underestimated by the patient, and no diagnostic procedure had been undertaken before the major episode of loss of consciousness. The present case underlines that, even in the presence of relatively vague complaints, an early diagnosis is essential to prevent major hypoglycemic episodes which can be potentially lethal both directly and by causing injury, as in motor vehicle accidents caused by loss of consciousness. In this setting, a proper and orderly diagnostic approach is essential to rule out the presence of an insulin-secreting tumor.

The appropriate diagnostic steps for a biochemical diagnosis of insulinoma include determination of circulating levels of insulin and C-peptide, and plasma insulin/glucose ratio. A suppression test is also usually needed. Although endogenous insulin production is normally suppressed in the setting of hypoglycemia, the lack of suppression during a 72-hour fast is a strong indicator of the presence of an insulin-secreting tumor. During the test, usually supervised in a hospital setting, capillary blood glucose is measured using a reflectance meter every 4 hours (and every hour when blood glucose is <60 mg/dL). When values are <50 mg/dL, or the patient has symptoms of hypoglycemia, a blood test is drawn for serum glucose, insulin, proinsulin, and C-peptide levels, and the fast is stopped.

Once a biochemical diagnosis of insulinoma is established, localization of the tumor is usually obtained with noninvasive imaging modalities.^8^ Although scintigraphy with ^111^In-octreotide is widely used for diagnosing somatostatin receptor positive tumors, it has a low sensitivity in detecting insulinomas, probably due to the lack of somatostatin receptors type 2 and the small size of the lesions.^8^ Whereas the sensitivity of transabdominal ultrasonography in the localization of insulinomas is poor (ranging from 9% to 64%), contrast-enhanced abdominal CT is currently accepted as the first-line investigation for the visualization of insulinomas. Insulinomas are typically hypervascular and, thus, demonstrate a greater enhancement than normal pancreatic parenchyma during the arterial and capillary phases of contrast bolus. The use of multidetector CT may increase the sensitivity of the technique.^9^ Magnetic resonance imaging is a suitable alternative to CT, has a similar or better sensitivity and specificity, and is probably the investigation of choice in defining hepatic metastases.^10^

In the case of small insulinomas not detected with the above noninvasive imaging modalities, invasive procedures may still be necessary to achieve preoperative localization. Endoscopic ultrasonography has a reported sensitivity of 87% to 92%, although it is largely operator dependent and its sensitivity is lower for insulinomas in the tail of the pancreas or extrapancreatic.^11^ Angiography combined with arterial stimulation venous sampling, using calcium as the insulin secretagogue, is probably the most sensitive available diagnostic technique, with an accuracy ranging from 94% to 100%.^12^ Manual palpation of the pancreas by an experienced surgeon and ultrasonography are both sensitive methods for the intraoperative detection of the site of insulinomas.

Surgical excision is the treatment of choice and is curative in most cases.^13^ After surgical therapy, patients with insulinomas generally have excellent long-term survival. Medical therapy with diazoxide, calcium channel blockers, Dilantin, and somatostatin is reserved for the cases which cannot undergo surgery.

Minimally invasive surgery for pancreatic diseases is a major new research field in abdominal surgery, which has been developed in the past 2 decades. The traditional laparoscopic approach is now widely used in the execution of a distal pancreatectomy for benign diseases, although there is still a lack of solid evidence.^14^ More controversial is the application of this approach in malignancies, in which the surgeon must ensure the same radical approach as the open technique. In the early 2000s, minimally invasive surgery underwent a great revolution with the introduction of robotic systems to overcome the limitations of traditional laparoscopy. The first case of robot-assisted distal pancreatectomy was described by Melvin et al in 2003.^15^

Technical limitations of laparoscopic pancreatic surgery include a restricted range of motion and the 2-dimensional operative field. Articulation of robotic instruments, physiological tremor suppression, 3D vision, and a stable image allow the surgeon to perform complex resection and anastomosis with the same techniques of the traditional open surgery.^16^ Another source of concern is represented by the costs of the procedure, although the increased accuracy and safety in tissue dissection may reduce postoperative complications and hospital stay, so decreasing overall costs.^17^

A recent systematic review of 5 nonrandomized studies on the topic^18^ showed some interesting aspects requiring further investigation. First, the procedure has a high feasibility, with a reported rate of conversion to open surgery of less than 5%. Major complications (Clavien grade III or IV) were only 0% to 5% among studies, with a pancreatic fistula rate of 15.4%. Overall, the review^18^ reported a 30-day postoperative morbidity between 0% and 18% and a 90-day postoperative mortality of 0% in all included studies. Perhaps, one of the most interesting aspects is the ability to perform a distal pancreatectomy with spleen preservation. In this respect, robotic surgery has a higher success rate than laparoscopy.^19^ A study by Kim et al^20^ confirms a considerably higher spleen-preserving rate in the robotic than in the laparoscopic group (*P* = 0.027).

However, we must emphasize that many different techniques have been reported in the literature, and standardization of the procedures is under way.^21^

## CONCLUSIONS

The case above highlights the complexity of the diagnosis of insulinoma when symptoms are nonspecific. It also underlines the importance of appropriately investigating for insulinoma hypoglycemic patients suffering or causing road traffic accidents. Robot-assisted distal pancreatectomy is a new treatment and there is still not enough data to draw final conclusions compared with conventional or laparoscopic surgery. In this case, the robotic approach allowed to achieve excellent results in terms of oncological outcomes, reduced postoperative pain, shorter hospital staying, early resumption of intestinal motility, and early oral nutrition, although the expected duration of the surgical intervention is usually longer than with laparoscopic or open surgery.
